# Spatial analysis of modern contraceptive use among women who need it in Ethiopia: Using geo-referenced data from performance monitoring for action

**DOI:** 10.1371/journal.pone.0297818

**Published:** 2024-04-04

**Authors:** Bedilu Alamirie Ejigu, Solomon Shiferaw, Paula Moraga, Assefa Seme, Mahari Yihdego, Addisalem Zebene, Ayanaw Amogne, Linnea Zimmerman

**Affiliations:** 1 Department of Statistics, Addis Ababa University, Addis Ababa, Ethiopia; 2 School of Public Health, Addis Ababa University, Addis Ababa, Ethiopia; 3 Computer, Electrical and Mathematical Sciences and Engineering Division, King Abdullah University of Science and Technology, Thuwal, Saudi Arabia; 4 PMA Ethiopia, Addis Ababa, Ethiopia; 5 Department of Population, Family and Reproductive Health, Johns Hopkins Bloomberg School of Public Health, Baltimore, MD, United States of America; Madda Walabu University, ETHIOPIA

## Abstract

**Introduction:**

The challenge of achieving maternal and neonatal health-related goals in developing countries is significantly impacted by high fertility rates, which are partly attributed to limited access to family planning and access to the healthcare systems. The most widely used indicator to monitor family planning coverage is the proportion of women in reproductive age using contraception (CPR). However, this metric does not accurately reflect the true family planning coverage, as it fails to account for the diverse needs of women in reproductive age. Not all women in this category require contraception, including those who are pregnant, wish to become pregnant, sexually inactive, or infertile. To effectively address the contraceptive needs of those who require it, this study aims to estimate family planning coverage among this specific group. Further, we aimed to explore the geographical variation and factors influencing contraceptive uptake of contraceptive use among those who need.

**Method:**

We used data from the Performance Monitoring for Action Ethiopia (PMA Ethiopia) survey of women of reproductive age and the service delivery point (SDP) survey conducted in 2019. A total of 4,390 women who need contraception were considered as the analytical sample. To account for the study design, sampling weights were considered to compute the coverage of modern contraceptive use disaggregated by socio-demographic factors. Bayesian geostatistical modeling was employed to identify potential factors associated with the uptake of modern contraception and produce spatial prediction to unsampled locations.

**Result:**

The overall weighted prevalence of modern contraception use among women who need it was 44.2% (with 95% CI: 42.4%—45.9%). Across regions of Ethiopia, contraceptive use coverage varies from nearly 0% in Somali region to 52.3% in Addis Ababa. The average nearest distance from a woman’s home to the nearest SDP was high in the Afar and Somali regions. The spatial mapping shows that contraceptive coverage was lower in the eastern part of the country. At zonal administrative level, relatively high (above 55%) proportion of modern contraception use coverage were observed in Adama Liyu Zone, Ilu Ababor, Misrak Shewa, and Kefa zone and the coverage were null in majority of Afar and Somali region zones. Among modern contraceptive users, use of the injectable dominated the method-mix. The modeling result reveals that, living closer to a SDP, having discussions about family planning with the partner, following a Christian religion, no pregnancy intention, being ever pregnant and being young increases the likelihood of using modern contraceptive methods.

**Conclusion:**

Areas with low contraceptive coverage and lower access to contraception because of distance should be prioritized by the government and other supporting agencies. Women who discussed family planning with their partner were more likely to use modern contraceptives unlike those without such discussion. Thus, to improve the coverage of contraceptive use, it is very important to encourage/advocate women to have discussions with their partner and establish movable health systems for the nomadic community.

## Background

The reduction of maternal and child mortality can be greatly achieved by increasing the accessibility and utilization of contraceptives, leading to a decrease in unintended pregnancies and unsafe abortions [1–3]. In East Africa, where maternal and child mortality rates are alarmingly high, contraceptive usage remains below 30% [4, 5]. Research conducted by Kasahun *et al* [6] indicates that expanding family planning services could notably contribute to achieving the SDG3 target of reducing maternal mortality. Moreover, enhancing contraceptive coverage may also play a role in alleviating poverty [7] and empowering women to make reproductive choices autonomously [8, 9].

The Federal Ministry of Health in Ethiopia has recognized family planning as a critical strategy for enhancing maternal and neonatal health [10]. Despite setting ambitious targets, the coverage of family planning has not progressed as anticipated [11]. Geographical disparities in the use of modern contraceptive methods and limited studies addressing factors linked with their uptake present significant challenges [12–14]. The identification of factors associated with the adoption of modern contraceptive methods and the examination of geographical variances in their usage are crucial for policymakers in devising targeted intervention strategies. However, the existing body of research on this subject is notably constrained for several reasons.

Firstly, estimates of the contraceptive prevalence rate (CPR) are generally computed by taking the percentage of all women in reproductive age (15 to 49 years old) who are using any type of contraception. This estimate, however, is not a true coverage indicator as not all women (i.e., pregnant women, menopausal women, women who wish to become pregnant) are in need of contraception [15]. Measuring progress towards Family Planning Coverage (FPC) would be strengthened by producing estimates for the proportion of women using contraception among those who need it [15, 16]. Thus, for program evaluation, FPC indicators respond more promptly to program changes (implementing intervention mechanisms) than impact indicators, such as CPR [16, 17]. Further details on the use and estimation techniques of FPC from CPR for monitoring and policy making were given by Barros *et al* [15]. To generate regional and national FPC indicators, this study focused on estimating contraceptive coverage among women in need of contraception method. The proposed indicator differs from the UN Sustainable Development Goal (SDG) indicator 3.7.1, “Proportion of women of reproductive age (aged 15-49 years) who have their need for family planning satisfied with modern methods” [18]. As highlighted by Barros et al [15] it’s important to note that the current UN SDG indicator 3.7.1 may not fully capture the true coverage of family planning. Hence, to better measure progress towards family planning coverage, it would be beneficial to produce estimates for the proportion of women using contraception among those who actually need it by explicitly excluding pregnant women, menopausal women, women who wish to become pregnant, sexually inactive and infecund women from the denominator. This would provide a more accurate assessment of family planning coverage and its impact.

Secondly, geospatial data are useful to make geographically targeted interventions for decision makers [19]. Mapping modern contraceptive use has great advantages for not only understanding where high/low coverage are happening, but also for understanding health facilities accessibility and human activity/practice in that area. Adding the spatial component of the data into the analysis have two advantages to program planners and decision makers: i) generating maps that are helpful to better understand the geographic context behind activities, and ii) geographic context can help facilitate linkages among data sets and wide diversity of health data, covering a wide range of policy and planning issues [19].

Thirdly, the use of modern contraception can be affected by both demand-side (individual level) and supply-side(health facility-level) factors. Despite this interdependence contraceptive research has tended to explore demand-side factor [5, 12, 13, 20–27], and supply-side factors separately. Studies done by [28–31] investigated both individual and service environment factors associated with modern contraceptive use, but their analytical samples were not women in need of a method, which may distort estimates.

A study by Solomon et al [32] used the 2016 EDHS data and the 2014 SPA data to explore the relationship between the service delivery environment and contraceptive use, but the linkage of data sources from two different years introduces substantial potential for bias. Further, the study by [12] defined access to the health facility by considering the average distance from sampled family planning providers to one point within a survey cluster, not to the sampled individual women house. This study considers distance from the nearest SDP to the exact household.

Previous studies on contraceptive use in Ethiopia have demonstrated the association of contraceptive use and education, parity, age, marital status, media exposure, household wealth quintile and place of residence, [13, 20–24, 33, 34], but few studies limit their analyses to women in need of contraception, and none explore this geographically. With these research gaps in mind, the objective of this paper is to explore the spatial distribution of modern contraceptive coverage and identify determinant factors that affect the uptake of modern contraception among women who need it. To the best of our knowledge, no study has examined the relationship between the use of modern contraception and the distance to the nearest health facility, restricting the sample to only women who have need of contraception. Findings from this study will provide evidence to inform decision-makers and stakeholders involved in family planning to improve women’s access to modern contraception.

The association between contraceptive use and various demographic factors in Ethiopia has been well-documented in previous studies. However, there is a lack of research focusing specifically on women in need of contraception, and none have explored this geographically. This paper aims to fill this gap by examining the spatial distribution of modern contraceptive coverage and identifying determinant factors that affect its uptake among women who need it. Additionally, this study will also investigate the relationship between the use of modern contraception and the distance to the nearest health facility. The findings of this research will provide valuable evidence to guide decision-makers and stakeholders in improving women’s access to modern contraception.

## Methodology

### Study design and data source

The data used in the study were obtained from Performance Monitoring for Action Ethiopia (PMA Ethiopia) project which includes a nationally representative survey of women of reproductive age and a service delivery point (SDP) survey, both conducted from October-December in 2019. The PMA surveys are cross-sectional surveys based on a multistage stratified cluster sampling design with urban-rural stratification. The primary sampling units are Enumeration Areas (EAs), selected using probability proportional-to-size methods. A total of 265 enumeration areas were selected by the Central Statistics Agency (CSA) to be representative at the national and regional level. Prior to data collection, all households in each enumeration area were listed and mapped by interviewers to create a frame for the second stage of the sampling process. Once listing was completed, 35 households per EA were randomly selected. All occupants in selected households were enumerated and from this list, all eligible women between the ages of 15 to 49 were administered the female-household questionnaire after informed consent was obtained. The female questionnaire collects information on Socio-demographic characteristics, reproductive preferences including birth history and contraceptive knowledge, use, history and intention. Additional details on PMA survey protocol, sampling design and implementation are further described by Zimmerman and colleagues [35].

The SDP survey is a combination of a census of governmental health facilities that serve the selected EA, specifically the health post, health center, and primary level hospital, and a sample of up to three private health facilities within the Kebele boundary (the lowest administrative level within Ethiopia). The SDP sample is thus representative of the service environment accessible to the female sample. The SDP questionnaire focuses on health facility characteristics, available services, staffing, infrastructure and the provision of family planning services including the availability of contraceptive methods, integration of services and observation of the exam room for family planning visits.

The advantage of using PMA data are two-fold. First, SDP and female data are collected at the same time, reducing biases introduced when analyzing data sources collected years apart, and second, GPS points were collected at the household and SDP allowing for individual level linkages. This is helpful to avoid miss-classification errors [36] associated with linking SDP data with the household data during the computation of the nearest distance from women household to the SDP.

### Analytical sample

Among a total of 8,837 women who completed the survey questionnaire, only 4,390 women who need contraception were considered. Women who are in-need of a contraceptive method defined as those who are sexually active, fecund, not pregnant and want to avoid pregnancy. This demographic represents a significant portion of the female population, and their access to effective contraception is essential for their well-being and autonomy. When discussing contraceptive needs, it is important to exclude certain groups from the analytical sample. Women who want to be pregnant, sexually inactive and those who are currently pregnant women were excluded from the analytical sample. As a result, the analysis was done only by considering 4,390 women.

### Study variables

The outcome variable of this study was modern contraceptive use. A woman was considered to be using modern contraception if she used any of the following methods; female sterilization, implant, IUD, injectable, pill, emergency contraception, female or male condoms, cycle beads, and LAM. A number of Socio-demographic characteristics, obstetric characteristics, distance to the nearest SDP and partner dynamics variables were considered as an exploratory variable. Table 1 presents summary of exploratory factors considered in this study.

**Table 1:** Description of exploratory variables considered in the study.

Variable	Variable description/classification
Age	Respondents age classified as: 15-19, 20-24, 25-34, 35-49
Education	Never attained, Primary, Secondary/higher
Wealth	Household Wealth quintiles (low, middle, high)
Parity	Number of births: 0-1 births, 2-3 births, 4+ births
Fertility intention	Have a/another child, No preference/Says she can’t get pregnant
Marital status	Not married, Married/in union, Single/divorced/widowed
Religion	Respondent religion: Christian, Muslim, Others
Discussion with Partner	Have discussion with the partner about family planning (Yes, No)
Partner feeling	How partner feel about FP (Disapprove, don’t care, ok with it)
Media^†^	Media Exposure about family planning
Residence	Place of residence: urban, rural
Region^‡^	List of administrative regions of Ethiopia
Zone	List of administrative zones of Ethiopia
Distance to SDP	Nearest distance to the SDP (<2Km, 2-6Km, >6Km)

^†^ It is a composite variable constructed using YES or NO question: did you heard/seen or received message about family planning from radio, TV, newspaper or magazine, social media, text message on mobile.

^‡^ Tigray, Amhara, Oromia, SNNP, Addis Ababa, Afar, Somali, B-Gumuz, Gambella, Harari,Diredawa. FP stands for Family Planning

### Statistical analysis

To investigate geographical disparity in modern contraceptive use and explore factors that influence the uptake of modern contraception, different spatial and non-spatial data analyses techniques were employed. The proportion of modern contraception users were computed by taking into account survey weights using STATA 17 software [37]. Graphical exploration and modeling was done using the **R** software [38].

#### Spatial analysis

Spatial analysis techniques offer several advantages over non-spatial modeling techniques, including the ability to incorporate geographic location, visualize data in a geographic context, and identify spatial dependencies. For these reasons, our study has employed a spatial data analysis approach in order to take full advantage of these benefits. Regional, Zonal and sampling cluster level geographic coordinates were used to map the coverage of modern contraceptive use under different administrative levels. Household level geographic coordinates were used when we perform spatial interpolation using model-based geostatistics [39].

#### Spatial interpolation

The spatial interpolation technique predicts modern contraceptive use coverage on the un-sampled areas in the country based on sampled data. To predict the minimum, average and maximum coverage of modern contraception use coverage we use a geostatistical model. Inference was performed using Integrated Nested Laplace Approximation (INLA) which is designed to perform approximate Bayesian inference in latent Gaussian models and implemented using the R-INLA package [40]. Prediction to the spatially continuous Gaussian random fields was done by solving Stochastic partial Differentiation Equation(SPDE) on a discrete mesh of points that covers Ethiopia using R-INLA [41].

Conditional on the true prevalence of modern contraceptive use *P*(*x*_*i*_) at location *x*_*i*_, *i* = 1, 2, …, *n*, the number of contraception users *Y*_*i*_ out of *n*_*i*_ women sampled follows a binomial distribution: *Y*_*i*_|*P*(*x*_*i*_) ∼ *Binomial*(*n*_*i*_, *P*(*x*_*i*_)),

$$\begin{array}{c} logit\left(P\left(x_{i}\right)\right)=\beta_{0}+D\beta +S\left(x_{i}\right) \end{array}$$ (1)

where, *β*_0_ denotes the intercept, *D* is a design matrix with rows corresponding to the covariate (Table 1) data and *S*(.) is a spatial random effect that follows a Gaussian process with mean zero, and variance *σ*^2^, and correlation function *ρ*(*u*) = *Corr*(*S*(*l*), *S*(*l*′)) [39]. Among different parametric families, such as exponential, Gaussian, spherical have been proposed for *ρ*(*u*), Stein [42] recommends the use of Matern correlation function [43] given by (Eq 2)

$$\begin{array}{c} \rho \left(u;\phi ,\kappa \right)=\frac{2^{\kappa -1}\Gamma \left(\kappa \right)}{{\left(u/\phi \right)}^{\kappa }\kappa_{\kappa }\left(u/\phi \right)},u>0 \end{array}$$ (2)

where *ϕ* > 0 is a scale parameter, *κ* > 0 is the shape parameter, *κ*_*κ*_(⋅) is the modified Bessel function of the second kind of order *κ* > 0, and *u* = ||*l* − *l*′|| is the Euclidean distance between two locations.

The predicted mean, lower and upper limits of 95% credible intervals of modern contraception use coverage across Ethiopia were done using Eq 1.

## Results

### Socio-demographic characteristics

In this analysis, 4,390 women in reproductive age (15-49 years old) who need contraception across 265 enumeration areas were included. Out of this number, 44.6% of them had no formal education, nearly quarter (26.3%) were between the age of 15-24, 83.1% were married, 38.1% were exposed to mass media about family planning, 68.5% were living ≤ 2km distance from any SDP, 65.2% had a child or have the intention to have another child, 69.6% of the respondents were living in rural areas of the country. Further, 40.2% were from a household with high wealth quintile, 43.0% had four and above children, 90.5% were ever pregnant, and 71.1% practiced christian religion. Further details of the respondents characteristics by different socio-demographic factors are presented in Table 2.

**Table 2:** Background characteristics of respondents.

Background Characteristics	Unweighted n	Weighted n	Percentage(weighted)
**Total**	**4390**	4390	100.0
**Age**			
15-19	326	354	8.1
20-24	820	799	18.2
25-34	1,804	1754	40.0
35-49	1,440	1482	33.8
**School**			
Never attend	1,789	1960	44.6
Primary	1,502	1551	35.3
Secondary/higher	1,099	879	20.0
**Marital Status**			
Not married	253	204	4.6
Married/Union	3,529	3649	83.1
Divorced/Widowed	608	537	12.2
**Religion**			
Chistian	3,153	3119	71.1
Muslim	1,155	1195	27.2
Others	82	76	1.7
**Wealth quantile**			
Low	1,452	1741	39.7
Middle	752	882	20.1
High	2,186	1767	40.2
**Parity**			
0-1 birth	1,382	1283	29.2
2-3 births	1,314	1221	27.8
4+ births	1,694	1886	43.0
**Distance from SDP**			
≤2 km	3,267	3008	68.5
2-6 Km	875	1163	26.5
> 6Km	248	219	5.0
**Media exposure**			
No	2,504	2714	61.9
Yes	1,883	1673	38.1
**Ever Pregnant**			
No	468	417	9.5
Yes	3,922	3973	90.5
**Partner discussion**			
No	2,646	2562	58.4
Yes	1,744	1828	41.6
**Partner Feeling**			
Disapprove	723	759	17.3
Don’t care	1,389	1220	27.8
Ok with it	2,278	2411	54.9
**Fertility Intention**			
Have a/another child	2,891	2862	65.2
No Preference	1,499	1528	34.8
**Residence**			
Urban	1,790	1334	30.4
Rural	2,600	3056	69.6
**Region**			
Tigray	554	265	6.0
Afar	149	31	0.7
Amhara	787	1025	23.4
Oromia	910	1741	39.6
Somali	67	112	2.6
B-Gumuz	159	57	1.3
SNNP	823	857	19.5
Gambella	207	17	0.4
Harari	198	20	0.5
Addis Ababa	368	244	5.6
Dire Dawa	168	21	0.5

### Health facility characteristics

During 2019 PMA-Ethiopia survey implementation, data from 799 service delivery points (health facilities) of which 547(68.5%) were public and 252(31.5%) were private facilities were collected. Among sampled SDPs 95.6% and 65.3% of them were providing family planning services and immediate postpartum family planning service, respectively. Further, 90.7% of the SDPs were providing family planning related counseling services.

Table 3 presents the summary of women’s home nearest distance from the SDP by region. The national average nearest distance from SDP to women’s home was 2.01km (with SD of ±4.38km). The longest minimum average distance from the facility to women’s homes were found in Afar (13.79km) region. Conversely, in Addis Ababa the average distance from women home to the SDP were 0.45km (with SD of ± 0.34km) (Table 3). Among the survey respondents, 74.42% and 5.65% were living ≤2km and >6km from the SDP, respectively. In the Afar region, majority (58.39%) of the respondents nearest distance from the SDP were above 6Km (Table 3).

**Table 3:** Summary of respondents nearest distance from the SDP by region.

Region	Summary of nearest distance from SDP					Percentage of women by nearest SDP		
	Mean	Median	Sd	min	max	<=2km	2-6Km	>6km
Tigray	1.52	0.79	2.16	0.02	12.12	81.41	10.83	7.76
Afar	13.79	8.31	14.88	0.13	50.33	30.87	10.74	58.39
Amhara	1.83	1.48	1.63	0.02	7.26	62.26	34.05	3.68
Oromia	2.22	1.23	4.43	0.01	28.67	66.92	28.35	4.73
Somali	2.06	0.63	3.34	0.12	15.91	76.12	11.94	11.94
B-Gumuz	1.87	0.89	2.18	0.07	7.99	70.44	18.87	10.69
SNNP	1.69	1.24	2.24	0.04	16.39	74.97	22.48	2.55
Gambella	0.94	0.39	1.41	0.03	5.81	88.89	11.11	0
Harari	0.89	0.41	1.03	0.02	4.47	86.36	13.64	0
Addis Ababa	0.45	0.34	0.29	0.01	1.31	100	0	0
Dire Dawa	0.56	0.51	0.36	0.06	1.72	100	0	0
**Total**	**2.01**	**0.88**	**4.38**	**0.01**	**50.33**	**74.42**	**19.93**	**5.65**

### Modern contraceptive use coverage

The coverage of modern contraceptive use among women who need it was found to be 44.2% of which 24.8% were injectable users, 14.2% implant users, 2.7% pills users, and 2.5% were other modern method users. The coverage in the use of modern contraception varied across administrative regions of the country; the highest modern contraception use among women who need it was reported in Addis Ababa (52.3%) followed by Amhara region (49.6%) and SNNP and Oromia regions (44.7%). As presented in Table 4, uptake of modern contraceptive methods were associated with different factors. The coverage of modern contraceptive uptake were 50.1% among women who have two-to-three children, 49.0% among married (in union) women, and 82.0% among women have had discussion about family planning with their partner in the past 12 months. Irrespective of different demographic factors (except region), among modern contraceptive users, injectibale users took the lion-share of the method-mix (Table 4). In our sample, women living in the Somali region are not using any of the modern methods. As compared with other modern contraception methods, women living in Addis Ababa, Dire dawa and Harari are implant users.

**Table 4:** The coverage of mCP by respondents background characteristics among women who need it.

Background Characterstics	mCPR	Method-mix			
		Injectables	Implant	Pill	Others
**Total**	**44.2**	**24.8**	**14.2**	**2.7**	**2.5**
**Age**					
15-19	45.5	29.7	11.9	2.5	1.3
20-24	52.9	30.0	15.4	4.0	2.9
25-34	49.2	27.5	16.3	3.1	2.6
35-49	33.2	17.6	11.6	1.6	2.5
**School**					
Never attend	35.5	21.0	12.1	0.9	1.5
Primary	49.8	28.1	16.2	3.3	2.5
Secondary/higher	53.8	27.3	15.3	5.6	4.7
**Marital Status**					
Not married	31.1	13.5	4.9	2.8	7.1
Married/Union	49.0	27.8	15.9	3.0	2.4
Divorced/Widowed	16.3	8.4	6.0	0.4	1.1
**Religion**					
Chistian	50.1	28.3	16.2	2.8	2.7
Muslim	28.5	15.6	8.7	2.3	1.8
Others	49.5	25.6	18.2	4.0	3.6
**Wealth quantile**					
Low	36.5	21.8	13.2	0.8	0.9
Middle	48.8	30.3	15.6	0.4	2.4
High	49.4	24.9	14.5	5.7	4.1
**Parity**					
0-1 birth	51.7	29.0	14.9	4.5	2.6
2-3 births	50.1	27.5	17.8	2.4	2.8
4+ births	35.2	20.1	11.3	1.7	2.2
**Distance from SDP**					
≤ 2 km	47.7	26.8	14.0	3.7	3.1
2-6 Km	38.9	21.4	15.7	0.6	1.2
> 6Km	23.9	14.5	8.5	0.0	0.9
**Media exposure**					
No	42.0	25.6	13.0	1.7	1.8
Yes	47.8	23.5	16.1	4.3	3.6
**Ever Pregnant**					
No	43.8	24.6	9.8	3.4	4.1
Yes	44.2	24.8	14.7	2.6	2.3
**Partner discussion**					
No	17.2	10.0	5.1	0.6	1.4
Yes	82.0	45.5	26.9	5.6	4.1
**Partner Feeling**					
Disapprove	26.4	14.6	8.2	1.0	2.6
Don’t care	22.7	12.3	6.5	1.0	2.2
Ok with it	60.6	34.3	20.0	4.1	2.6
**Fertility Intention**					
Have a/another child	48.4	27.7	15.2	3.2	2.1
No preference	36.3	19.3	12.2	1.8	3.2
**Residence**					
Urban	51.8	24.9	15.3	6.8	4.7
Rural	40.9	24.7	13.7	0.9	1.5
**Region**					
Tigray	36.7	17.6	15.7	1.6	1.7
Afar	2.2	1.3	0.0	0.8	0.0
Amhara	49.6	32.8	12.6	2.6	1.5
Oromiya	44.7	24.0	15.7	2.3	2.8
Somali	0.0	0.0	0.0	0.0	0.0
Benishangul-Gumuz	44.0	25.2	15.8	2.1	0.8
SNNP	44.7	26.7	13.6	2.6	1.9
Gambella	44.4	27.7	11.5	3.7	1.5
Harari	27.4	5.7	12.0	3.9	5.4
Addis Ababa	52.3	14.7	18.6	8.7	8.6
Dire Dawa	34.3	7.3	16.0	4.6	7.5

### Spatial distribution of modern contraceptive use coverage

**Fig 1:**
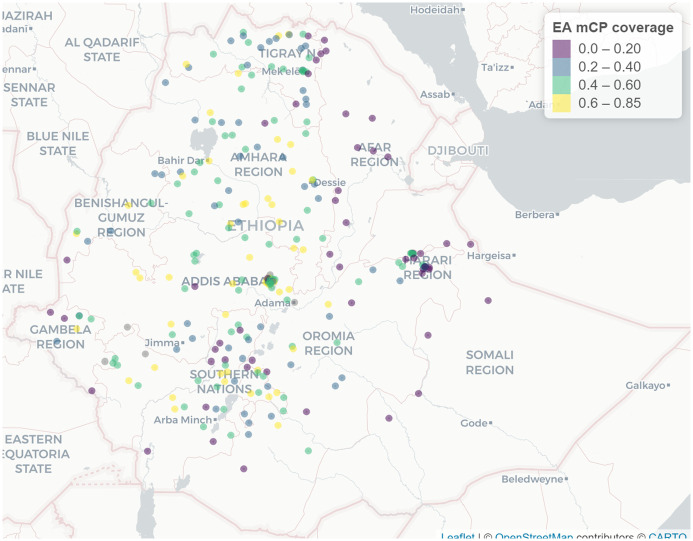
Cluster level distribution of modern contraceptive use coverage in Ethiopia, PMA 2019 (Source: Shapefile obtained from UN OCHA Ethiopia https://data.humdata.org/organization/ocha-ethiopia?).

The cluster level spatial analysis result reveals that there is a spatial clustering in the distribution of modern contraceptive users among women who need it in Ethiopia (Global Moran’s *I*=0.27, Z-score = 7.74, p-value <0.0001). The hot-spot areas with relatively high coverage (yellow color) of modern contraceptive use were located in the Western and Central part of the country (Fig 1).

**Fig 2:**
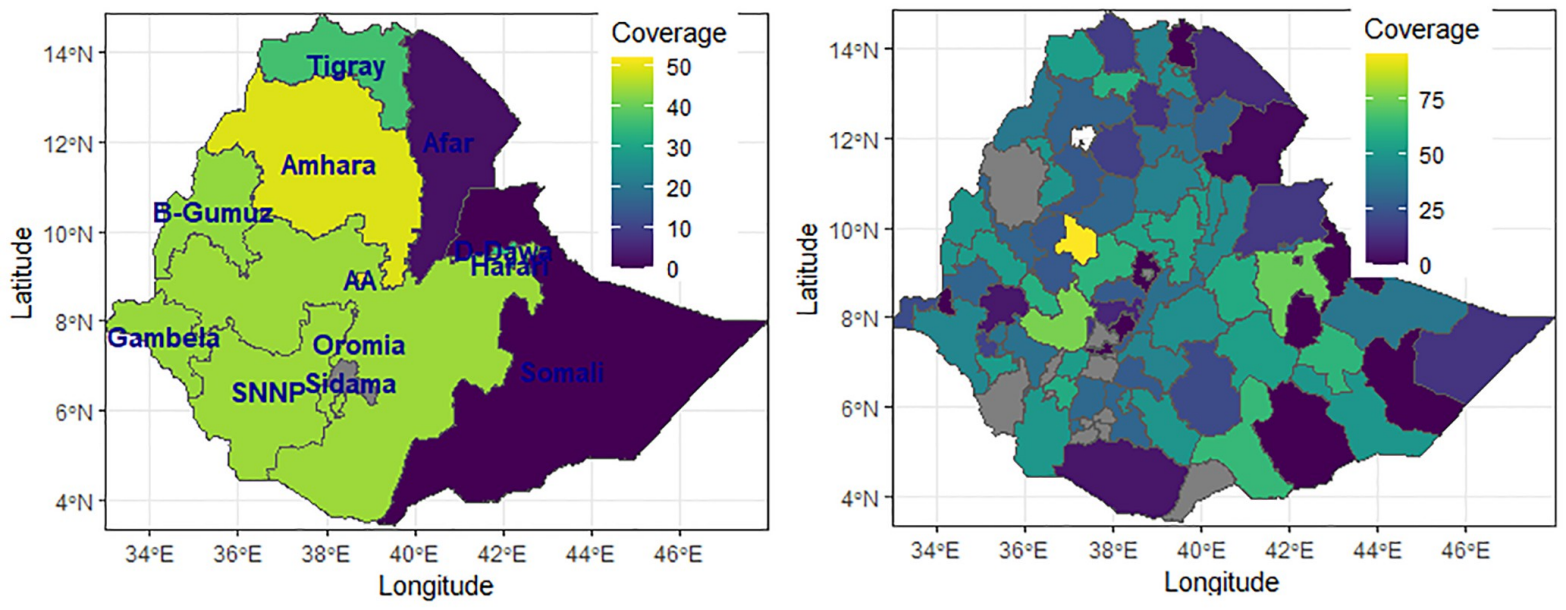
Regional (left panel) and Zonal(right panel) level distribution of modern contraceptive use coverage among women who need it in Ethiopia, PMA 2019 (Source: Shapefile obtained from UN OCHA Ethiopia https://data.humdata.org/organization/ocha-ethiopia?).

In addition to cluster level spatial exploration, the Zonal and Regional level coverage maps of modern contraceptive use were generated to show relatively high and low coverage areas (Fig 2). The result shows the coverage was relatively high in the western and central parts of the country, and it was lower in the eastern part.

### Spatial prediction of modern contraceptive use coverage

**Fig 3:**
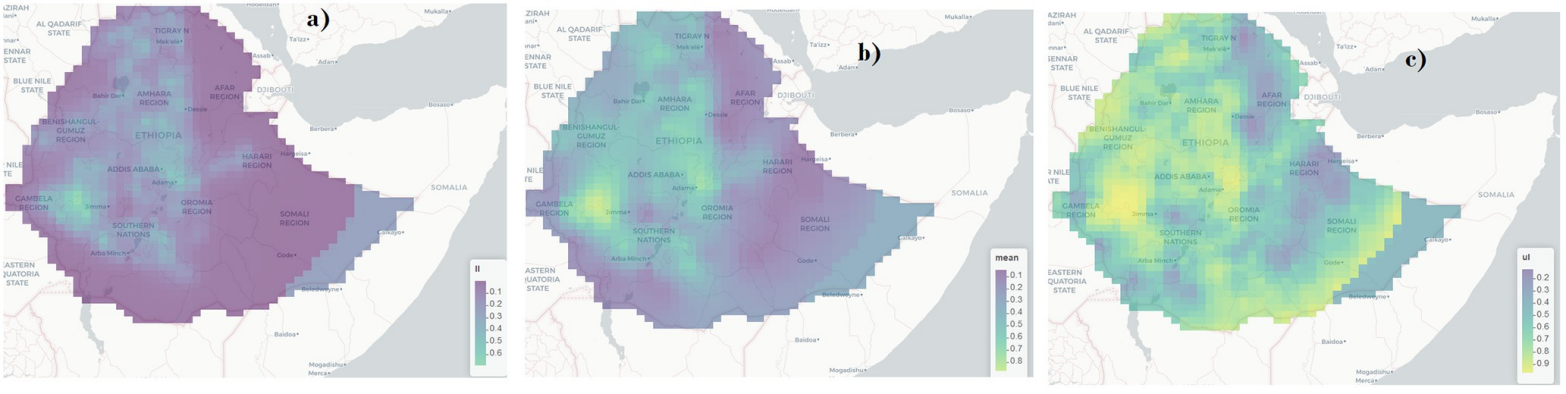
Map of modern contraceptive coverage prediction in Ethiopia: a) 2.5% quantile, b) Posterior predictive mean and c) 97.5% quantile of the coverage of modern contraceptive use (Source: Shapefile obtained from UN OCHA Ethiopia https://data.humdata.org/organization/ocha-ethiopia?).

Fig 3 presents the predicted mean, and lower and upper limits of 95% credible intervals of modern contraception use coverage across the country. The prediction was done using model 1. The result reveals that the coverage of modern contraception use was lower in the eastern part of the country and relatively high in the north-west and central part of the country. The prediction results depicted on the map allow us to see the distribution of modern contraception use coverage in Ethiopia and helpful to understand which areas have a high or low coverage so appropriate policy action can be taken to improve the coverage in the uptake of modern contraception.

### Bayesian geostatistical modeling results

Table 5 presents summary of Bayesian geostatistical modeling results. Note that, the coefficients of an INLA model can be interpreted in the same way as the coefficient of a logistic regression model. But, since INLA is a Bayesian modeling approach, rather than single estimates estimates are described using probability distributions [39]. The modeling result reveals, the likelihood of using modern contraception among women of reproductive age who need contraception was high among those who were from high wealth quintile household (Posterior Odds Ratio (POR)=1.52, 95%CI (1.12,2.06)), women who had discussion with the partner about family planning (POR = 20.25, 95%CI (16.54, 24.88)), compared to women who were those from low household wealth quintile, and who were not discussed about FP with their partner. Women who were 35 years and above(POR = 0.59,95%CI (0.38, 0.93)), those practicing Muslim (POR = 0.57, 95%CI (0.43, 0.75)), those who were divorced/widowed(POR = 0.39, 95%CI (0.0.26, 0.59)) and those who attend secondary/higher education (POR = 0.59, 95%CI (0.44, 0.79)) were less likely to use modern contraception as compared with the reference groups (Table 5).

**Table 5:** Summary of the Bayesian geostatistical modeling results.

Factor	Posterior Estimates				Posterior Odds Ratio (POR)			
	Mean	Sd	95% CI		Mean	Sd	95% CI	
**Age (ref:15-19)**								
20-24	-0.04	0.19	-0.42	0.33	0.96	1.21	0.66	1.40
25-35	-0.05	0.20	-0.45	0.35	0.95	1.22	0.64	1.42
36-49	-0.52	0.23	-0.98	-0.07	0.59	1.26	0.38	0.93
**School(ref:Never attained)**								
Primary	0.04	0.11	-0.18	0.27	1.04	1.12	0.83	1.31
Secondary/higher	-0.53	0.15	-0.82	-0.23	0.59	1.16	0.44	0.79
**Marital Status(ref:Single)**								
Married/union	-0.02	0.21	-0.43	0.40	0.99	1.23	0.65	1.49
Divorced/widowed	-0.94	0.21	-1.35	-0.52	0.39	1.23	0.26	0.59
**Religion(ref:Christian)**								
Muslim	-0.57	0.15	-0.85	-0.28	0.57	1.16	0.43	0.75
Others	0.33	0.38	-0.43	1.07	1.38	1.46	0.65	2.92
**Wealth (ref:Low)**								
Middle	0.31	0.14	0.05	0.58	1.37	1.14	1.05	1.78
High	0.42	0.16	0.11	0.72	1.52	1.17	1.12	2.06
**Residence(ref:Urban)**								
Rural	0.00	0.15	-0.29	0.30	1.00	1.16	0.75	1.34
**Parity(ref:0-1 births)**								
2-3 births	-0.18	0.13	-0.44	0.08	0.84	1.14	0.64	1.09
4+ births	-0.30	0.17	-0.63	0.04	0.74	1.18	0.53	1.04
**Media (ref:No)**								
Yes	0.11	0.10	-0.08	0.30	1.12	1.10	0.92	1.35
**Ever pregnant(ref:No)**								
Yes	0.19	0.19	-0.19	0.56	1.21	1.21	0.83	1.75
**Partner discussion(ref: No)**								
Yes	3.01	0.10	2.81	3.21	20.25	1.11	####	24.88
**Partner(ref:disapprove)**								
Don’t care	0.12	0.17	-0.22	0.45	1.12	1.18	0.81	1.57
Ok with it	-0.09	0.14	-0.37	0.19	0.91	1.15	0.69	1.21
**Fertility intention (ref:Yes)**								
No	0.01	0.12	-0.22	0.23	1.01	1.12	0.80	1.26
**SDP distance(ref:≤2Km)**								
2-6 Km	-0.26	0.14	-0.53	0.01	0.77	1.15	0.59	1.01
> 6 Km	-0.62	0.30	-1.22	-0.04	0.54	1.35	0.30	0.96
**Region(ref:Addis Ababa)**								
Tigray	0.58	0.56	-0.52	1.70	1.79	1.75	0.60	5.45
Afar	-1.59	0.86	-3.37	0.00	0.20	2.36	0.03	0.99
Amhara	1.11	0.50	0.14	2.10	3.03	1.65	1.15	8.17
Oromia	0.61	0.37	-0.13	1.33	1.83	1.45	0.88	3.79
B-Gumuz	0.84	0.65	-0.41	2.15	2.32	1.92	0.66	8.61
SNNP	0.93	0.48	-0.01	1.87	2.54	1.61	0.99	6.48
Gambella	0.13	0.63	-1.09	1.36	1.13	1.87	0.34	3.91
Harari	0.29	0.67	-1.01	1.64	1.34	1.95	0.36	5.16
Dire dawa	0.39	0.66	-0.94	1.70	1.48	1.94	0.39	5.45
Spatial effects								
*θ*_1_	-2.36	0.36	-3.07	-1.65	-2.36	0.36	-3.07	-1.65
*θ*_2_	1.51	0.56	0.81	2.21	1.51	0.56	0.812	2.21

A woman who traveled a minimum of 6km to get a health facility was 46% less likely to use modern contraceptive methods compared with those who had access to health facilities within 2km. As compared with women residing in others regions of the country, women living in Afar (POR = 0.20, 95%CI (0.03, 0.99)) region was less likely to use modern contraception. The posterior odds ratio of modern contraception use among women who need it increased with increasing household wealth quintile.

Table 6 presents the percentage points difference between most- and least-advantaged socioeconomic subgroups in modern contraception use. The result shows that there was 99 percentage points difference by SDP accessibility between women who have access to SDP within 2km and those who get access to SDP after traveling a minimum of 6km. Further, there was 35% percentage points difference in the use of modern contraceptive use among women from high wealth quintile group as compared with women from the low wealth quintile.

**Table 6:** Absolute difference between the most- and least-advantaged socioeconomic subgroups in modern contraception use (percentage points).

Factor	Between the most- and least-advantaged Subgroups		
	Comparison	% difference	95%CI
Wealth quintile	High Vs low	35	(15, 56)
SDP accessibility	≤2km Vs >6km	99	(6, 193)
Education	Secondary/higher Vs not attend	51	(30, 73)
Residence	Urban Vs Rural	27	(9, 44)

**Fig 4:**
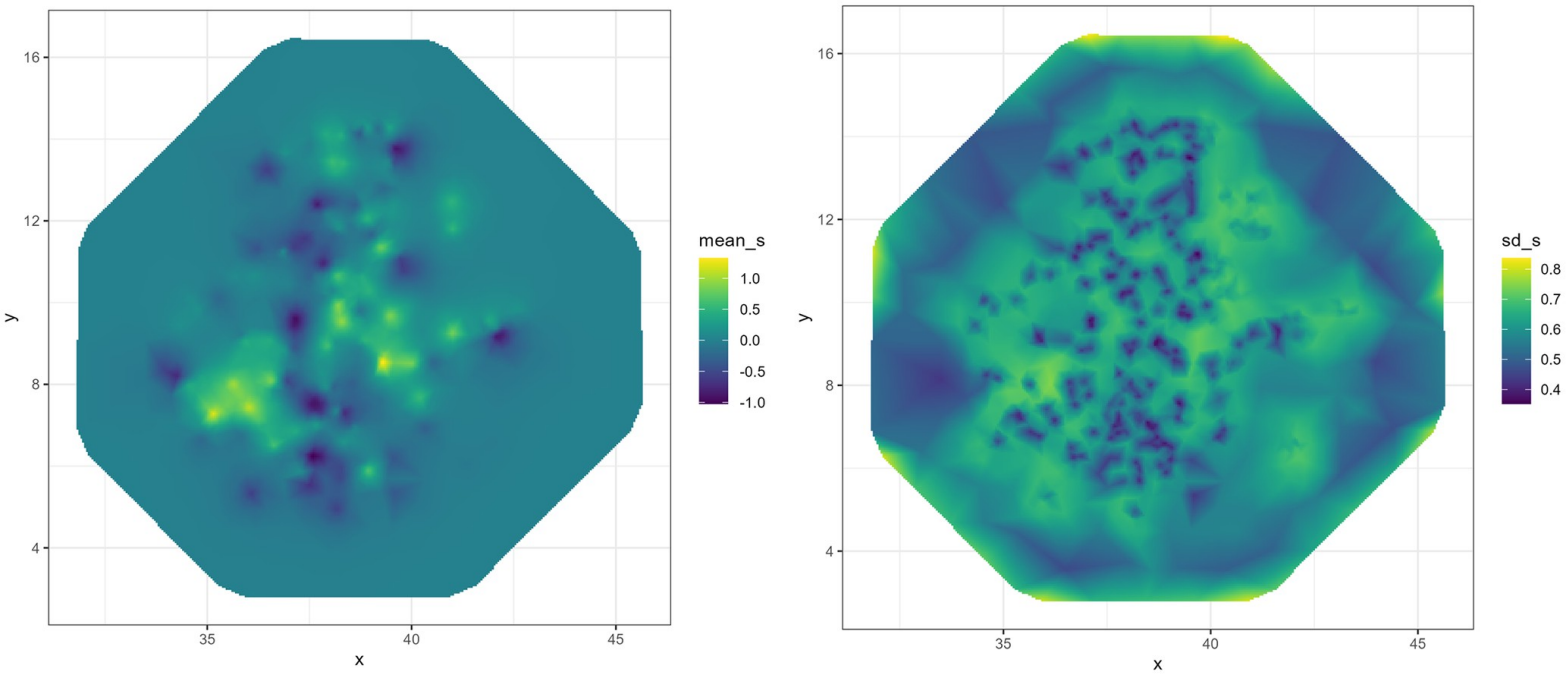
Mean and standard deviation of the spatial field across our mesh.

Fig 4 presents the mean and SD of the spatial field across the mesh. The figure shows that the mean of the spatial field across the mesh is non-uniform which reveals that there is spatial effect on the coverage of modern contraception use that we not accounting for with the factors included in our model. Thus, in areas where we see high standard deviation we require more data to make an accurate prediction of the true value of modern contraception use coverage.

In summary, in this Section the coverage of modern contraceptive use among women who need it in Ethiopia were discussed. The coverage varies across administrative regions, with the highest coverage reported in Addis Ababa. The use of modern contraception is associated with different factors, such as the number of children and discussion with a partner about family planning. Injectable users are the most common method of modern contraception. Further, we present geostatistical modeling results, which show that women from high wealth quintile households and those who had discussed family planning with their partners were more likely to use modern contraception. Women who were 35 years and above, Muslim, divorced/widowed, and those with secondary/higher education were less likely to use modern contraception.

## Discussion

This study examines the spatial distribution of modern contraception use coverage and identifies factors contributing to the uptake of contraception among women who need it, found that coverage of modern contraceptive use is very low(< 5%) in the eastern part of the country and women living near to the SDP are more likely to use modern contraception. Understanding geographical variation and identifying determinants of contraception use plays a major role in designing effective interventions that leads to increased use of modern family planning methods. This study found substantial significant variation in use of modern FP methods across different administrative regions of the country. A relatively higher proportion of modern contraception use was located in the central and western part of the country, and a low proportion of modern contraceptive use was observed in the eastern part of the country. Specifically, inline with previous studies [12, 20, 21, 24], this study found that women living in the eastern part of the country were less likely to use modern contraception than those women residing in other regions. This may be due to the difference in socio-cultural practices and beliefs [44]. For example Afar and Somali region populations are pastoralists and nomadic which may limit them to easily access health facilities to get family planning methods. Another reason for this finding may be women residing in Afar and Somali regions are less educated [45] and low wealth quintile [46]. Further, this may be linked with less access to health facilities [12] in these two regions.

The coverage of modern contraception use was high (>45%) in Addis Ababa and Amhara region. In line with other study findings [12, 32, 47], the uptake of modern contraceptive use is higher in women who are living near to the health facility.

The percentage of method-mix distribution among total contraceptive use across various methods-reflects both supply and demand (client preferences). When we see the method-mix distribution, among modern contraceptive users, injectibale users took the lion-share (Table 4). Even though, injectibales are the predominant methods, implant users are relatively high in Addis Ababa, Harari and Dire Dawa. In our study sample, there were no women from Somali region who are using any of the modern contraception methods (Table 4). Since method-mix can often be considered as a proxy for method availability and choice [48], it is very important to further investigate the availability of different contraceptive methods in areas where one method dominates the method mix, especially in Somali region. In addition to other factors, women who discussed family planning with their partner were more likely to use modern contraceptives, unlike those without such discussion. Thus, to improve the coverage of modern contraceptive use, it is very important to encourage/advocate women to have discussions with their partner.

Similar with other study findings [4, 13, 25, 49], Muslim women were less likely to use modern contraception as compared with Christians. The study done by [50–52] found that there is significant evidence for a relationship between religious socialization and contraceptive behavior. Heaton [51] examined religious group differences in fertility in 30 developing nations and found that Muslim fertility is substantially higher than Christian fertility in many countries.

In line with other studies [12, 13, 25, 32] the likelihood of using modern contraception increased with increasing wealth quintile. Women who were living in the high quintile wealth group were 52% more likely to use the service compared with those in the low quintile group. Access to contraception affected women’s economic outcomes through their educational attainment, labor force participation and career outcomes [53]. A study by [54] suggests that investing in family planning is a development “best buy” that can accelerate achievement across the five SDG themes. Thus, in collaboration with partner agencies increasing the coverage of modern contraception use especially in areas with low modern contraception use coverage is essential.

Similar with the findings [4, 25], we found that divorced/widowed women were less likely to use modern contraception methods as compared with unmarried/single women. Women who were 35 years old and above were less likely to use modern contraception compared to those in younger ages. This finding is similar with other studies [12, 13, 25]. This may be due to the fact that women aged 35 and above want to give birth compared to their younger counterparts [12].

Following the four FP2030 Ethiopia government commitments [55] to achieve Ethiopia’s Family Planning 2030 Vision, the ministry of health is working to increase access and ensure availability of quality family planning information and service. As studied by [3], investing in contraceptive service greatly reduces the need for public spending on health and other social services. The low level coverage of contraceptive use leads to high level of unintended pregnancy which ultimately becomes a cause of unsafe abortion [56].

### Strength and limitations of the study

This study has several advantages. First, the use of nationally representative data boosts the capacity of our findings to be generalized to women in Ethiopia. Further, employing spatial data analysis techniques enabled us to identify low- and high-coverage modern contraceptive use in Ethiopia, which will benefit both program designers and implementers in their design on context-specific and population-targeted interventions to enhance modern contraceptive use. Nevertheless, the results presented in this study should be considered in light of some limitations. One of the major limitations was that the data used in this study obtained from a cross-sectional survey, limiting us from establishing causality. Further, the data was self-reported, making it highly susceptible to recall bias and social desirability bias. Moreover, covariates considered in this study were limited to those available in the survey.

### Policy and practical implications

Findings from this study have implications for policy recommendation and practice. Women in the eastern part of the country should be given critical attention in family planning education programs. More efforts should be invested in providing family planning access to women without fertility intentions in these regions, as this will further increase the rate of modern contraceptive use among women who need it in Ethiopia. Given the observed result in the modern contraceptive use, women residing in the Somali region are not being reached by current family planning programs. Findings from our study has the following implications for policy and practice.

1. Integrating trained, equipped and supported health extension workers with the nomadic and pastoralists community, especially in Afar and Somali regions.
2. Expansion of SDPs and freely avail FP methods to create physical and economic accessibility.
3. Arrange mobile outreach services to provide a wide range of contraception.

## Conclusion and recommendation

The study found that the coverage of modern contraceptives has substantial geographic disparity in Ethiopia ranging from nearly 0% to 52.3% across the country, with significant regional variations. Significant disparity was also observed by religion, distance from the nearest health facility, fertility intention, partner support and discussion about family planning, wealth quintile, educational attainment, age and marital status. The findings of this study have several implications; first regions and zones with low contraceptive coverage should be targeted for scaling up family planning services, second based on the largest nearest distance to the SDP, it is important to launch additional health service facilities to ensure equity of access to health facilities. Third, increasing educational opportunities for those who practiced Muslim region is important to increase the coverage in Somali and Afar region. Further,to improve the coverage of contraceptive use, it is very important to encourage/advocate women to have discussions with their partner and establish movable health systems for the nomadic community.

## Abbreviations

EDHS: Ethiopian Demographic Health survey
INLA: Integrated Nested Laplace Approximation
POR: Posterior Odds Ratio
SD: Standard Deviation
SPA: Service Provision Assessment
